# Ameliorative effect of recombinant human lactoferrin on the premature ovarian failure in rats after cyclophosphamide treatments

**DOI:** 10.1186/s13048-020-00763-z

**Published:** 2021-01-21

**Authors:** Shubin Li, Mengnan Liu, Hongmeng Ma, Qin Jin, Yuzhen Ma, Chunyu Wang, Jingyu Ren, Gang Liu, Yanfeng Dai

**Affiliations:** 1 College of Life Science, Inner Mongolia University, 235 West University Road, Hohhot, 010021 Inner Mongolia China; 2 Centre of Reproductive Medicine, Inner Mongolia Hospital, 20 Zhaowuda Road, Hohhot, 010021 Inner Mongolia China; 3 Clinical Medicine Research Center, Chifeng municipal hospital, 1 Zhaowuda Road, Chifeng, 024000 Inner Mongolia China; 4 Department of Pathology, Affiliated Hospital of Nantong University, 20 Xisi Road, Nantong, 226001 Jiangsu China; 5 Key Laboratory of Medical Cell Biology, Clinical Medicine Research Center, the Affiliated Hospital of Inner Mongolia Medical University, 1 Tongdao North Street, Hohhot, 010050 Inner Mongolia China

**Keywords:** rhLF, POF, Folliculogenesis, ROS production, Ovarian apoptosis

## Abstract

This study investigated the effect of recombinant human lactoferrin (rhLF) on the premature ovarian failure (POF) of rats. After cyclophosphamide treatments, the POF rats were divided into the following groups: normal control group (NC), low-dose group (LD), medium-dose group (MD) and high-dose group (HD) of rhLF. After drug administrations, the ovarian indexes and hormonal levels were detected. After follicle number count, the proliferation and apoptosis were analyzed with the expressions of genes related with oogenesis, reactive oxygen species (ROS) production and apoptosis detected, followed by the calculation of oxidative stress and protein expressions. After 4-hydroperoxy cyclophosphamide (4-HC) treatments, the effect of rhLF on the proliferation, ROS production and gene expressions of primary rat granulosa cells (GCs) cultured in vitro were detected. After mating, the fertilities of POF rats were recorded. The result showed that the rhLF administrations up-regulated the ovarian index with the number of developing follicles increased and the decreases of hormonal levels conferred. The Ki-67 intensities of the MD and HD groups were up-regulated with the Tunnel intensities decreased. The rhLF treatments significantly promoted the expression of oogenesis, antioxidant and anti-apoptosis related genes. The expression of Bax and Caspase 3 were decreased with the expression of Bcl-2 up-regulated after rhLF administrations. The in vitro treatments of rhLF effectively conferred the toxicity of 4-HC on primary rat GCs. The fertility assessment showed the rhLF treatments up-regulated the offspring’s’ folliculogenesis, which confirmed the ameliorative role of rhLF on the POF damages via the inhibition of ROS production in GCs.

## Introduction

During the past decades, the incidence and mortality of female reproductive tumors, including cervical carcinoma, endometrial carcinoma and ovarian carcinoma have been rapidly growing worldwide [3]. The complicated causes and recurrences of these malignant tumors have been formidable threats to women’s well-beings. Although more efficient methodologies for tumor screening, diagnosis, surgery and treatments have been developed in recent years, the clinical outcomes and prognosis of female reproductive tumor patients remain to be further improved [35].

Nowadays, these current therapies for the surgery and radiotherapy have been the primary opinions for the clinical treatment of local and non-metastatic tumors, also, the anti-cancer therapies including chemotherapy, targeted therapies, hormone and biological therapies are the promising choices for the treatment of metastatic tumors [52]. However, due to the cellular toxicity, multidrug resistance (MDR) and reproductive disorders during the long term medication of anti-cancer drugs, the present application of anti-cancer drugs and recovery after drug administration still need to be further optimized for the development of more-effective personalized therapies [9, 10, 54].

Among these numerous chemotherapy drugs targeting mitochondria activities [including apoptotic related proteins, cytochrome c, caspase 3, caspase 9 and reactive oxygen species (ROS)] [1, 69, 77], endoplasmic reticulum stress (including inositol-requiring enzyme 1α, PKR-like ER kinase and activating transcription factor 6α) [8], nucleus (including nucleolar phosphorprotein, nuclear pore complex and nuclear localization signal) [28, 64], tumor microenvironments [26, 41, 71] and plasma membrane phospholipids [20, 31, 32], cyclophosphamide (CTX), as an orally active alkylating agent, has been widely used as an utilized antineoplastic drug for the clinical treatment of ovarian, breast, testicular and hematological tumors [16].

However, the clinical applications of CTX have been reported with the oxidative stress-induced toxicities in vivo [29, 45, 51, 56, 57, 62], which further resulted in the irreversible damages of germ cells, cellular apoptosis, infertility and premature ovarian failure (POF). Different protective agents isolated from herbal and antioxidants reducing oxidative stress have been developed to prevent the reproductive toxicity of CTX [19]. Furthermore, lactoferrin (LF) has been reported with the suppression abilities of oxidative stress-induced toxicities and cellular apoptosis [17, 38, 42, 49, 50].

As a natural pleiotrophic glycoprotein from the transferrin family, mammalian LF is mainly produced by epithelial cells, neutrophil precursors and placenta [7]. The presence of mammalian LF in these biological fluids including tears, saliva, pancreatic fluid, nasal, semen, urine and mostly abundant in mammalian milk further regulated the following biological processes as iron transport, anti-microbial defense and immune system regulation [34, 60, 67, 74]. And the large scale manufacture of bovine LF from skim milk and whey has been established from the 1990s [7]. In 2006, bovine LF has been applied as supplements to cosmetics, beverages, cosmetics, infant formula, pet foods and yogurt in Japan [66], meanwhile, the commercial infant formulas enriched with bovine LF has been available in Indonesia, South Korea and Spain [7].

In addition, due to the protective properties of antioxidant and anti-cancer, recombinant human lactoferrin (rhLF) modulates the production of cytokines with regards to cancer progression [21, 27, 68].

However, the therapeutic effect of rhLF on the mammalian reproductive abilities after CTX treatments via the suppression of oxidative stress-induced toxicities and cellular apoptosis remains unclear. Therefore, the aim of this study was to investigate the potential effects of oral rhLF administration on the therapeutic effect of rat POF-damages caused by CTX treatments. After the treatment of 4-hydroperoxy cyclophosphamide (4-HC, as the active in vitro metabolite of CTX), the effect of rhLF treatments on the proliferation, ROS production and gene expression levels of primary rat granulosa cells (GCs) cultured in vitro were detected to further confirm the ameliorative effect of rhLF on the POF damages and provide further basis for the clinical applications of rhLF.

## Results

### The physiological states of CTX-induced POF rats after rhLF administrations

First of all, the body weight of each rat after CTX treatments was examined to ensure the availabilities of the following experimental data and the results showed there were no significant differences among the body weight of 80 rats after CTX treatments.

**Fig. 1:**
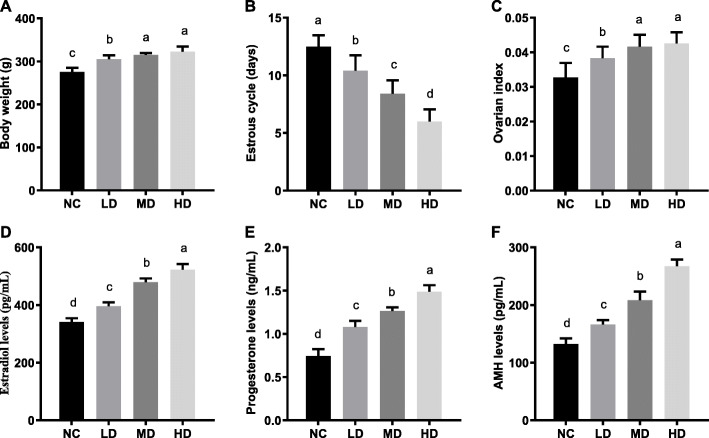
The physiological states of CTX-induced POF rats after rhLF administrations. **a** The body weight of CTX-induced POF rats in different groups after rhLF administrations; **b** The estrous cycle of CTX-induced POF rats in different groups after rhLF administrations; **c** The ovarian index of CTX-induced POF rats in different groups after rhLF administrations. **d** The estradiol levels of CTX-induced POF rats in different groups after rhLF administrations; **e** The progesterone levels of CTX-induced POF rats in different groups after rhLF administrations; **f** The AMH levels of CTX-induced POF rats in different groups after rhLF administrations. Note: In each panel, labeling with the different letter in each column indicates significant differences between different groups (*P* < 0.05)

As shown in Fig. 1a, the body weight of rats in all rhLF administration groups were remarkably higher in comparison with the NC group (275.38 ± 9.61) (*P* < 0.05), meanwhile, there was no significant difference between the body weight of the MD (315.07 ± 4.44 g) and HD groups (322.63 ± 11.96 g).

As shown in Fig. 1b, the abnormalities of estrous cycles in rats caused by CTX treatments were significantly reverted by rhLF administrations in a dose-dependent manner (*P* < 0.05). Furthermore, the results of ovarian index analyses showed that the ovarian indexes in all rhLF administration groups were significantly increased (Fig. 1c) in comparison with the NC group (*P* < 0.05), which indicated that the rhLF administrations significantly ameliorated the abnormal estrous cycles and improved the ovarian development of CTX-induced POF rats.

Furthermore, the hormonal levels of estradiol, progesterone and AMH in rats from different groups were analyzed to investigate the effect of rhLF administrations on the secretion of reproductive hormones. As shown in Fig. 1d, the results of estradiol level analyses showed that the estradiol levels of rats were significantly increased from 341.26 ± 12.73 pg/mL for the NC group, 395.80 ± 13.66 pg/mL for the LD group, 479.01 ± 13.26 pg/mL for the MD group to 522.28 ± 20.09 pg/mL for the HD group (*P* < 0.05).

In addition, the progesterone levels of rats in the rhLF administration groups were significantly higher than the NC group (0.74 ± 0.08 ng/mL), meanwhile, the progesterone levels of rats in the rhLF administration groups were positively correlated with the concentration of rhLF administration (*P* < 0.05) (Fig. 1e).

Furthermore, the AMH levels of rats in the rhLF administration groups significantly increased from 132.26 ± 9.81 pg/mL for the NC group, 166.31 ± 7.35 pg/mL for the LD group, 208.44 ± 14.93 pg/mL for the MD group to 267.32 ± 11.68 pg/mL for the HD group (*P* < 0.05).

These above results further confirmed that the rhLF administrations improved the abnormal secretion of reproductive hormones in a dose dependent manner, suggesting that the rhLF administrations could significantly ameliorated the physiological disorders of CTX-induced POF rats.

### The histological analyses of ovaries from CTX-induced POF rats after rhLF administrations

To further analysis the effect of rhLF administrations on the physiological state of ovaries from CTX-induced POF rats, the histological analyses of HE were conducted, followed by follicle number count.

**Fig. 2:**
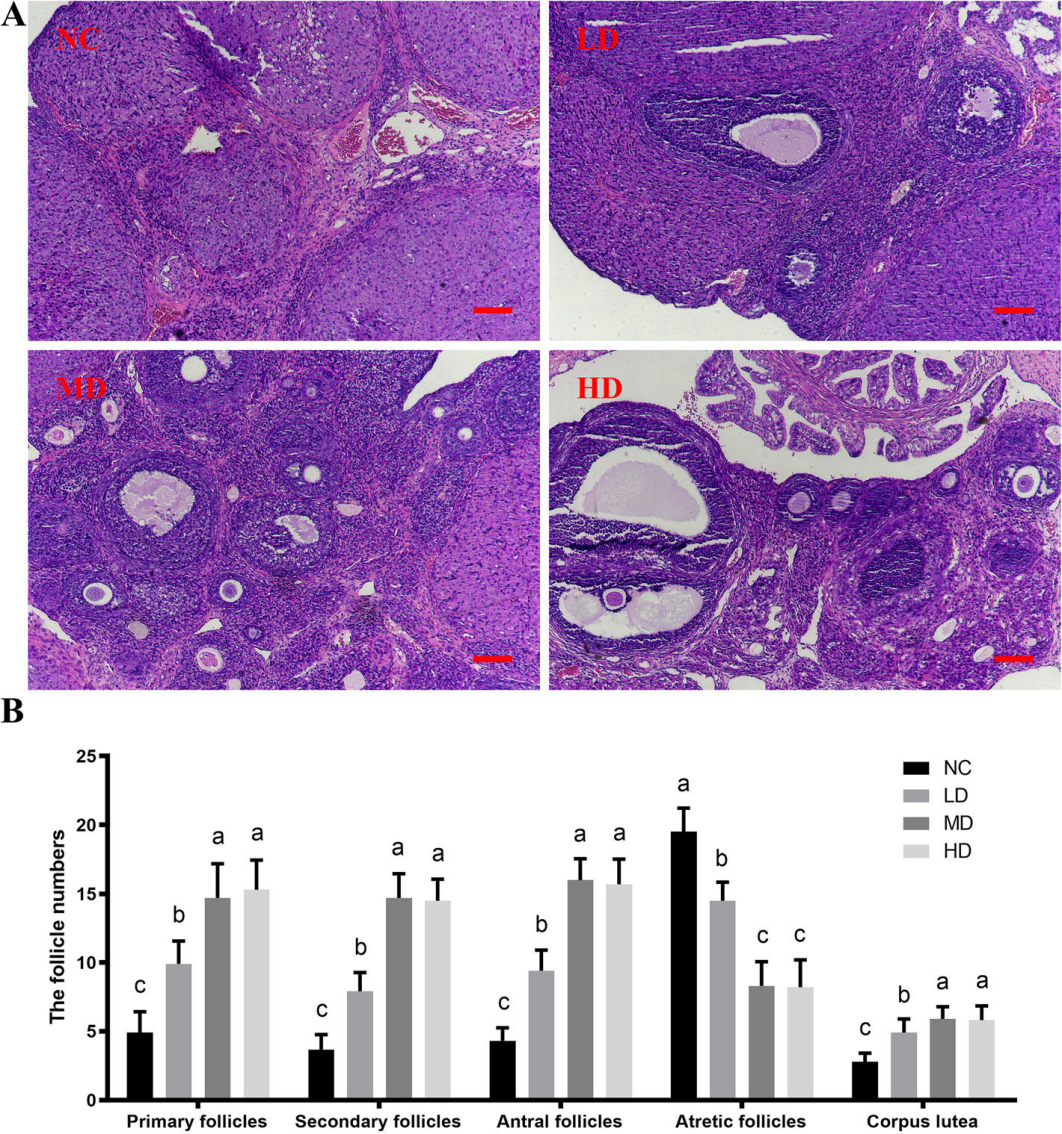
The histological analyses of ovaries from CTX-induced POF rats after rhLF administrations. **a** The representative HE staining results of ovaries from CTX-induced POF rats in different groups after rhLF administrations; Scale bar =100 μm; **b** The follicle number of ovaries from CTX-induced POF rats in different groups after rhLF administrations. Note: In each panel, labeling with the different letter in each column indicates significant differences between different groups (*P* < 0.05)

As shown in Fig. 2a, an abnormal histology with ovarian interstitial fibrosis, inflammatory cell infiltration and vessel dilation was observed in the rat ovaries of the NC group. The histological results showed that after rhLF administrations, the ovarian damages caused by CTX treatments were significantly alleviated in the rat ovaries.

And the results of follicle number count showed that the number of developing follicles at different stages (primary, secondary and antral follicles) in the ovaries of rhLF administration groups was significantly increased in comparison with the NC group (*P* < 0.05), however, the number of atretic follicles in the ovaries of the NC group was significantly higher than all rhLF administration groups (*P* < 0.05, Fig. 2b). Meanwhile, there were no significant differences between the number of developing follicles (regardless of primary, secondary or antral follicles) in the MD and HD groups. In addition, the number of corpus lutea in all rhLF treatment groups was significlantly up-regulated in comparison with the NC group (*P* < 0.05, Fig. 2b).

### The proliferative abilities of follicles from CTX-induced POF rats after rhLF administrations

**Fig. 3:**
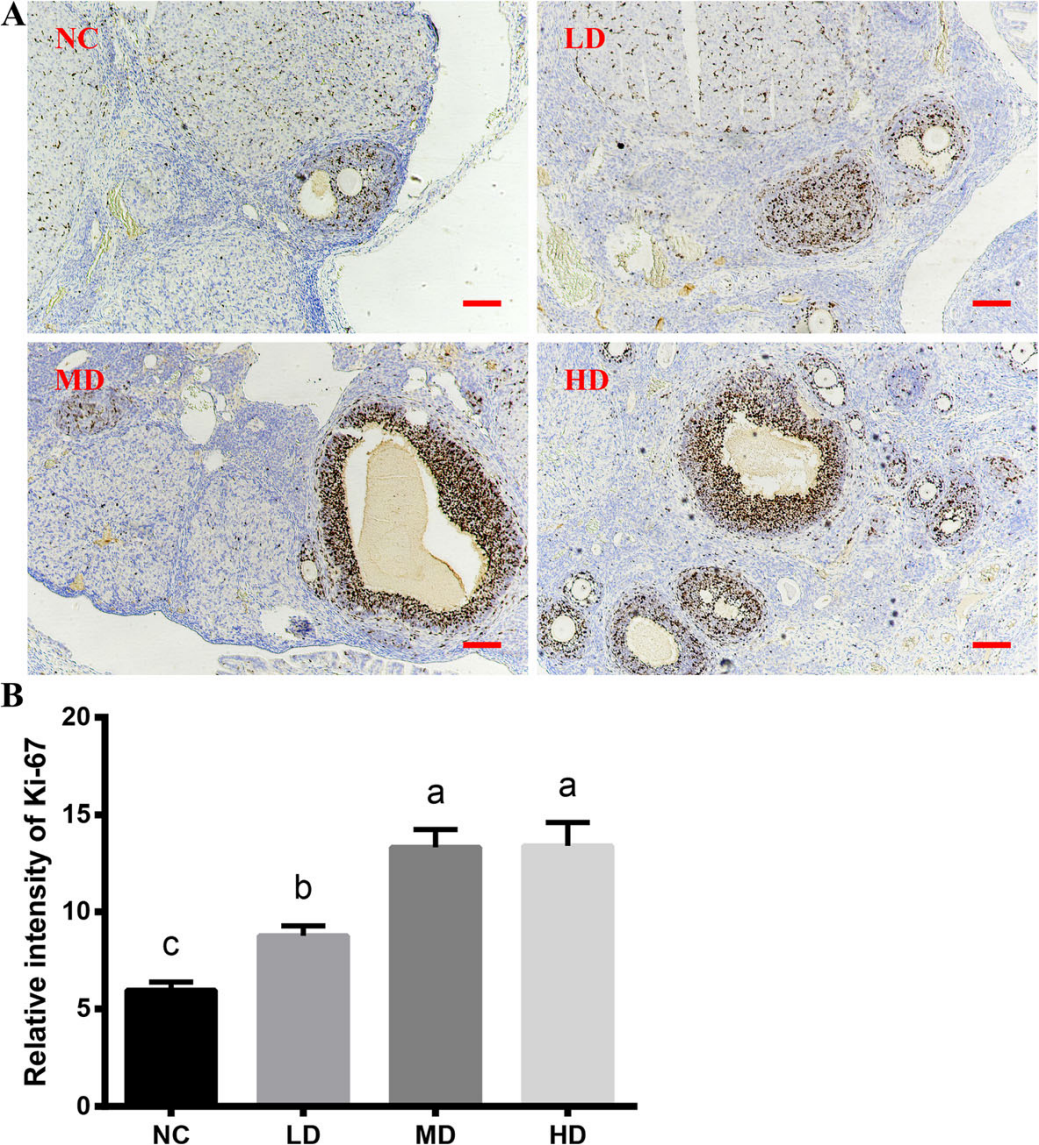
The Immunohistochemical staining results of ovaries from CTX-induced POF rats after rhLF administrations. **a** The representative Immunohistochemical staining of Ki-67 of ovaries from CTX-induced POF rats in different groups after rhLF administrations. Scale bar = 100 μm. **b** The relative expression levels of Ki-67 of ovaries from CTX-induced POF rats in different groups after rhLF administrations. Note: In each panel, labeling with the different letter in each column indicates significant differences between different groups (*P* < 0.05)

As shown in Fig. 3, the Immunohistochemistry (IHC) staining results of Ki-67 protein showed that the positive intensities of Ki-67 (mainly detected in GCs) in all rhLF administration groups were significantly higher compared with that of the NC group (*P* < 0.05), in addition, there was no significant difference between the Ki-67 positive intensities of the MD group and HD groups, which further confirmed that the rhLF administrations significantly promoted the proliferations of ovarian cells after CTX treatments.

### The apoptosis levels of follicles from CTX-induced POF rats after rhLF administrations

**Fig. 4:**
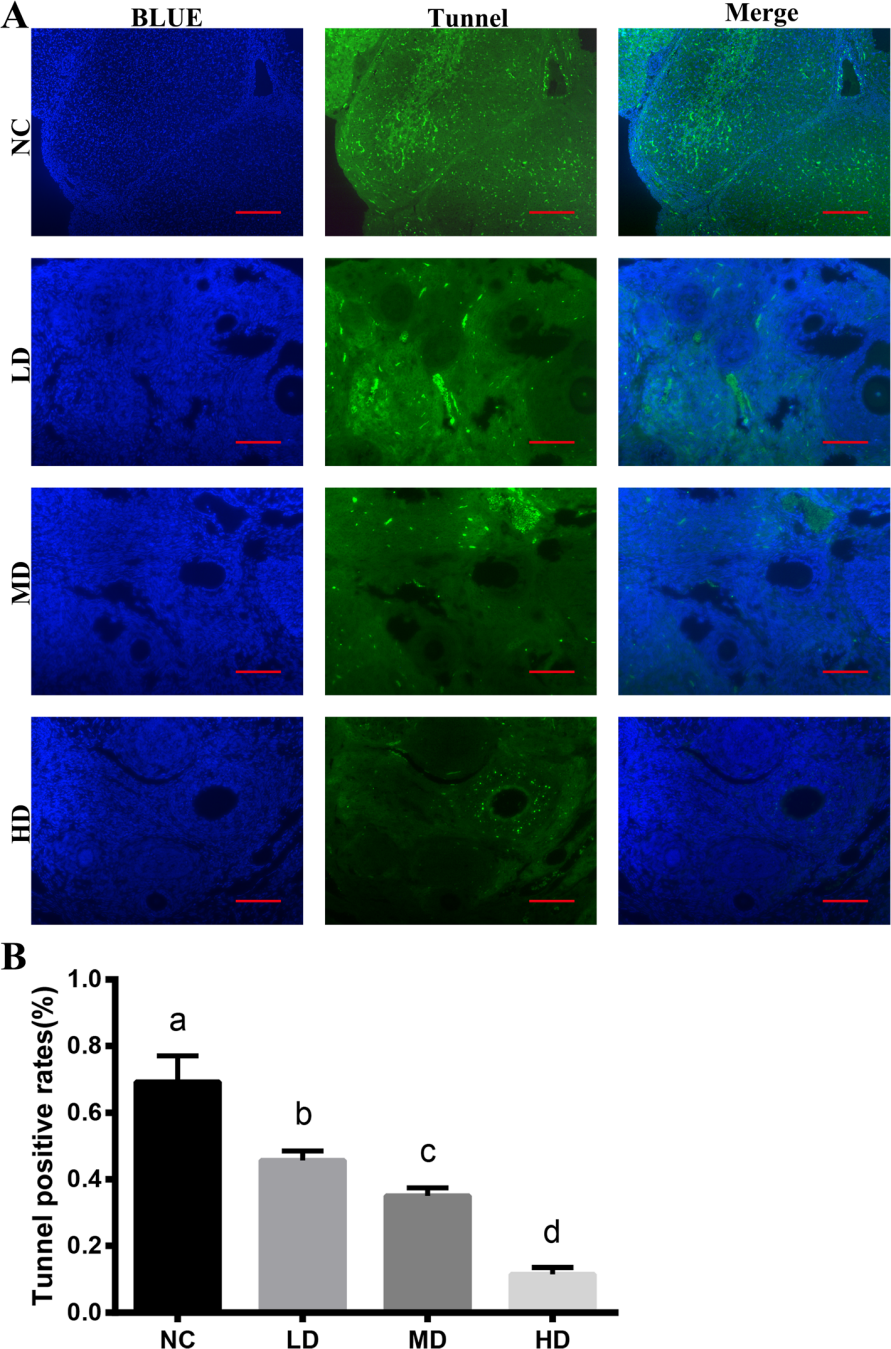
The Tunnel staining results of ovaries from CTX-induced POF rats after rhLF administrations. **a** The representative Tunnel (grenn) and DAPI (blue) staining results of follicles from CTX-induced POF rats in different groups after rhLF administrations; Scale bar = 200 μm; **b** The relative Tunnel rates (%) of follicles from CTX-induced POF rats in different groups after rhLF administrations. Note: Labeling with the different letter in each column indicates significant differences between different groups (*P* < 0.05).

Tunnel staining was further conducted to investigate the effect of rhLF administrations on the follicular apoptosis levels. As presented in Fig. 4, the majority of apoptosis cells mainly located in GCs, furthermore, the Tunnel rates as cellular apoptosis rates (%) in the rhLF administration groups were significantly reduced in comparison with the NC group (*P* < 0.05).

### The antioxidant abilities of ovaries from CTX-induced POF rats after rhLF administrations

**Fig. 5:**
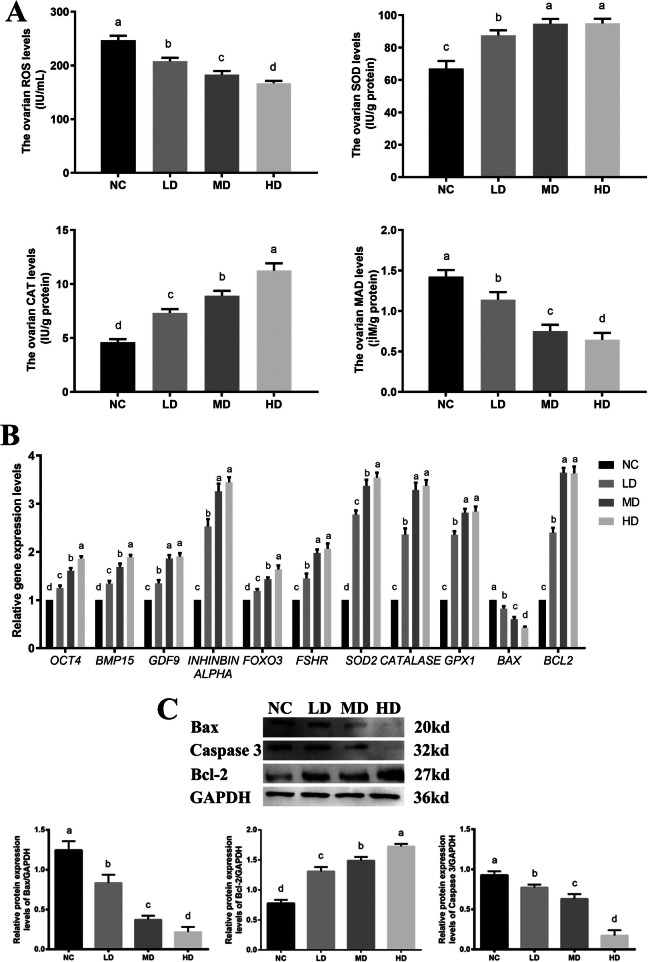
The antioxidant abilities of CTX-induced POF rats after rhLF administrations. **a** The ovarian ROS production levels (IU/mL), SOD levels (IU/g), TAOC levels (IU/g) and MDA levels (μM/g) in different groups after rhLF administrations. **b** The relative gene expression levels in ovaries from CTX-induced POF rats after rhLF administrations. **c** The protein expression patterns in ovaries from CTX-induced POF rats after rhLF administrations. Note: In each panel, labeling with the different letter in each column indicates significant differences between different groups (*P* < 0.05).

As shown in Fig. 5a, the results of ovarian ROS production levels showed that the ovarian ROS production levels were significantly reduced from 247.01 ± 8.30 IU/mL for the NC group, 207.69 ± 6.48 IU/mL for the LD group, 182.52 ± 7.01 U/mL for the MD group to 166.90 ± 4.29 U/mL for the HD group in a dose dependent manner (*P* < 0.05). Furthermore, the rhLF administration significantly promoted the ovarian SOD levels from 66.97 ± 4.77 IU/g for the NC group, 87.56 ± 3.18 IU/g for the MD group, 94.74 ± 2.93 IU/g for the LD group to 94.98 ± 2.80 IU/g for the HD group (*P* < 0.05). The ovarian CAT levels in all rhLF administration groups (as 7.32 ± 0.35 IU/g for the LD group, 8.91 ± 0.45 IU/g for the MD group and 11.25 ± 0.67 IU/g for the HD group) were significantly higher in comparison with the NC group (4.61 ± 0.28 IU/g) (*P* < 0.05). The ovarian MDA level of the NC group (1.42 ± 0.08 μM/g) was significantly higher than the LD (1.14 ± 0.09 μM/g), MD (0.75 ± 0.08 μM/g) and HD groups (0.64 ± 0.09 μM/g), respectively (*P* < 0.05).

The results of RT-PCR showed that the transcription levels of these specific genes for oogenesis as *Oct4*, *Bmp15* and *Gdf9* were significantly increased in all rhLF administration groups in a dose dependent manner (*P* < 0.05, Fig. 5b), which was similar with the expression patterns of these specific genes of GCs as *Inhibin alpha, Foxo3* and *Fshr* in all rhLF administration groups. Furthermore, the expression levels of these specific genes for the antioxidant abilities as *Sod2*, *Catalase* and *Gpx1* were significantly increased after rhLF administrations, which further confirmed the antioxidant characteristic of rhLF on POF ovaries after CTX treatments (*P* < 0.05). After rhLF administrations, the ovarian expression levels of *Bax* was significantly decreased in comparison with the NC group (*P* < 0.05). Meanwhile, the ovarian expression levels of *Bcl-2* in all rhLF administration groups were significantly higher than the NC group (*P* < 0.05), however, there was no significant difference in the expression level of *Bcl-2* between the MD and HD groups.

To further confirm the effect of rhLF administrations on the ovarian apoptosis process, the ovarian expression levels of Bcl-2, Bax and Caspase 3 were detected by Western blot. As shown in Fig. 5c, significantly decreased levels of Bax and Caspase 3 were found in all rhLF administration groups compared with the NC group (*P* < 0.05), meanwhile, the expression levels of Bcl-2 in all rhLF administration groups were significantly higher than the NC group (*P* < 0.05), which further confirmed that the rhLF administrations significantly inhibited the ovarian apoptosis process.

These above results further indicated that the rhLF administration significantly inhibited the ovarian oxidative stress levels and enhanced the ovarian antioxidant abilities after CTX treatments.

### The ROS production, proliferation and gene expression levels of primary rat GCs after 4-HC and rhLF treatments

**Fig. 6:**
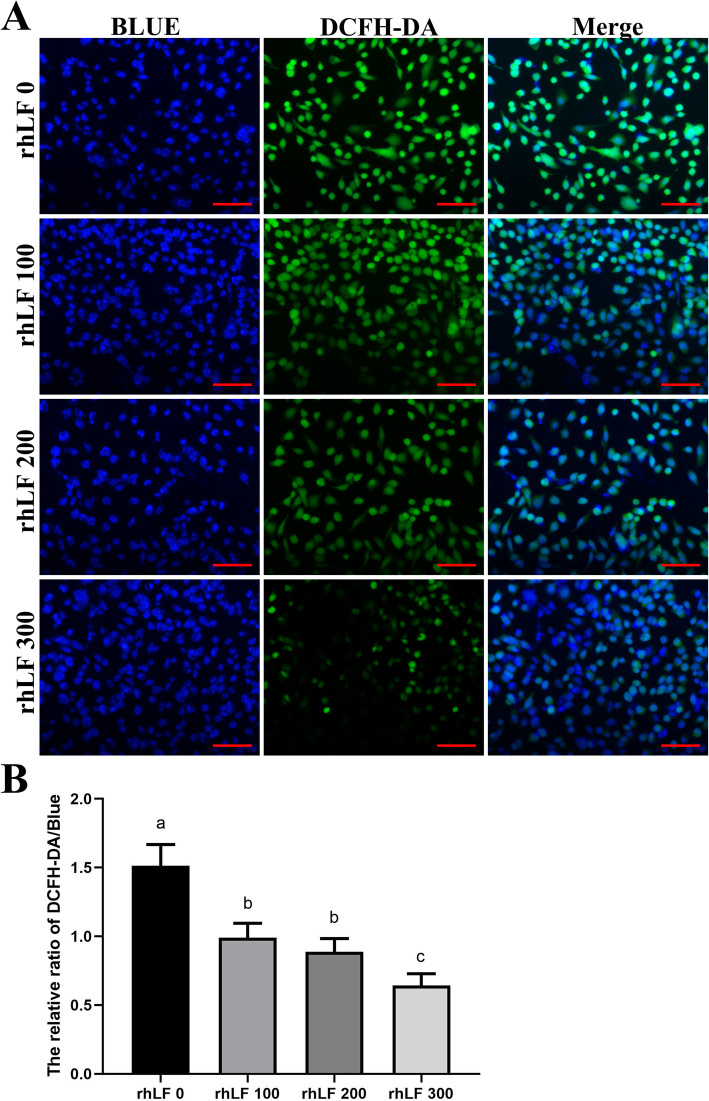
The ROS staining results of primary rat GCs after 4-HC and rhLF treatments. **a** The representative ROS staining results of primary rat GCs in different groups after rhLF treatments. Scale bar = 100 μm. **b** The relative densities of ROS staining of primary rat GCs in different groups after rhLF treatments. Note: In each panel, labeling with the different letter in each column indicates significant differences between different groups (*P* < 0.05)

To confirm the effect of rhLF treatments on the ROS production levels of primary rat GCs, ROS staining of primary rat GCs were analyzed with the representative images of DCFH-DA staining shown in Fig. 6a. As shown in Fig. 6a, the fluorescence intensities of DCFH-DA in primary rat GCs were significantly reduced in all rhLF groups compared with the rhLF 0 group (*P* < 0.05).

**Fig. 7:**
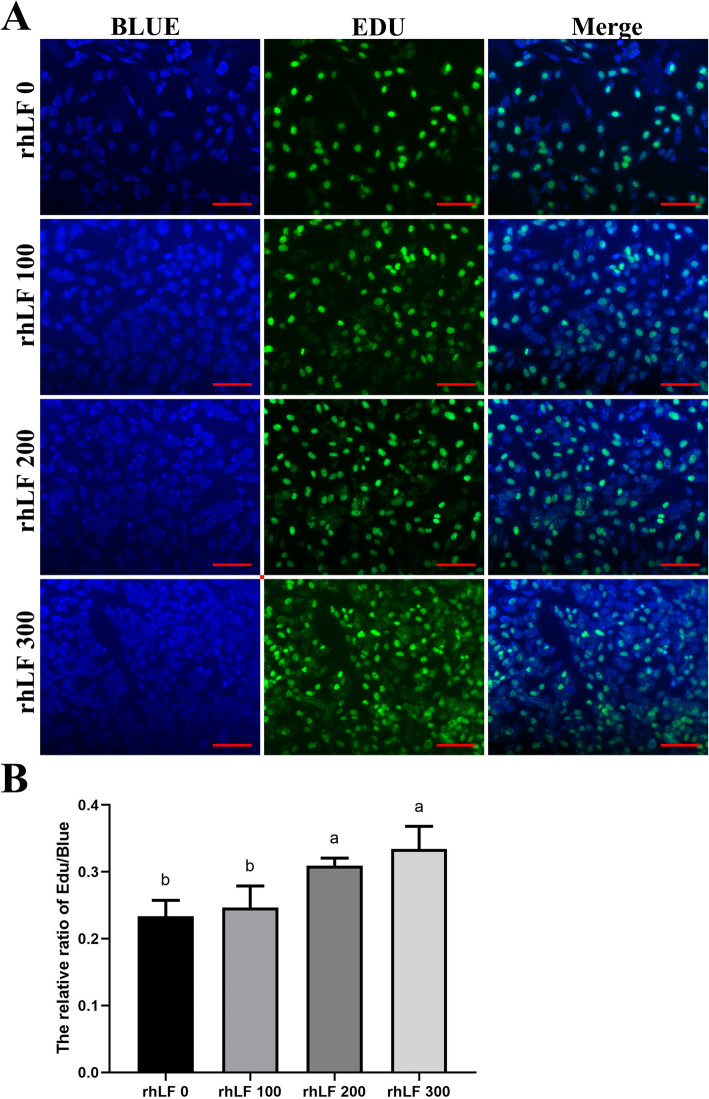
The EDU staining results of primary rat GCs after 4-HC and rhLF treatments. **a** The representative EDU staining results of primary rat GCs in different groups after rhLF treatments. Scale bar = 100 μm. **b** The relative densities of EDU staining of primary rat GCs in different groups after rhLF treatments. Note: In each panel, labeling with the different letter in each column indicates significant differences between different groups (*P* < 0.05)

Furthermore, the fluorescence intensities of Edu staining in primary rat GCs were significantly enhanced in the rhLF 200 and rhLF 300 groups, compared with the rhLF 0 and rhLF 100 groupS (*P* < 0.05) (Fig. 7), indicating that the treatment of rhLF significantly inhibited the ROS production levels and promoted the proliferative potentials of primary rat GCs during the in vitro treatment of 4-HC.

**Fig. 8:**
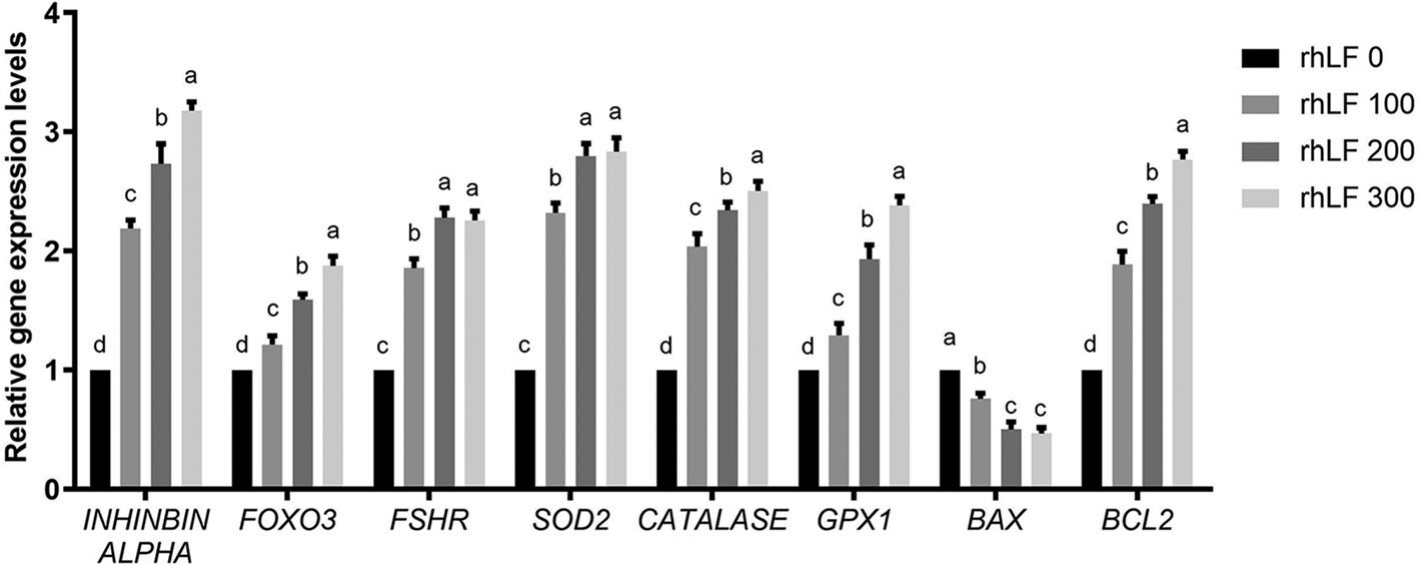
The gene expression levels of primary rat GCs after 4-HC and rhLF treatments. Note: In each panel, labeling with the different letter in each column indicates significant differences between different groups (*P* < 0.05)

In addition, the RT-PCR results of primary rat GCs during the in vitro treatments of 4-HC and rhLF confirmed that the rhLF treatments significantly up-regulated the cellular expression levels of *FOXL2*, *Inhibin alpha*, *SOD2*, *Catalase*, *GPx* and *Bcl-2* in a dose dependent manner (Fig. 8, *P* < 0.05). On the other hand, the expression levels of *BAX* in the NC group were significantly higher than that of all rhLF groups (*P* < 0.05).

### The fertility abilities assessment of CTX-induced POF rats after rhLF administrations

To further confirm the effect of rhLF administrations on the fertility abilities of CTX-induced POF rats, the assessments of fertility abilities were conducted. As shown in Table 1, the results showed that the rhLF administration resulted in a significant increase in the litter sizes in comparison with the NC group (*P* < 0.05), meanwhile there was no significant difference in the litter sizes among all rhLF administration groups.

**Table 1:** The fertility index and litter sizes in different groups after rhLF administrations

	NC	LD	MD	HD
Mating index	10/10(100%)	10/10(100%)	10/10(100%)	10/10(100%)
Fertility index	7/10(70%)	10/10(100%)	10/10(100%)	10/10(100%)
Mean litter size	5.67 ± 1.80b	14.22 ± 1.56a	14.44 ± 1.33a	15.33 ± 1.32a

**Fig. 9:**
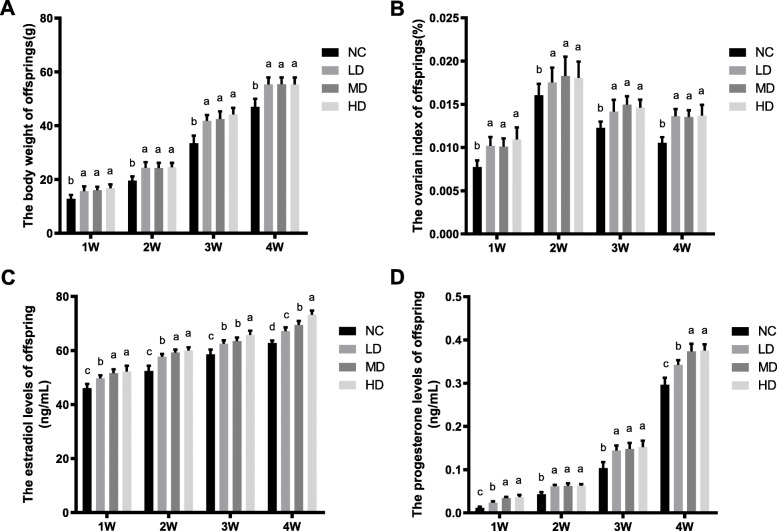
The physiological states of offspring from CTX-induced POF rats after rhLF administrations. **a** The body weight of offspring from CTX-induced POF rats after rhLF administrations; **b** The ovarian index of offspring from CTX-induced POF rats after rhLF administrations; **c** The estradiol levels of offspring from CTX-induced POF rats after rhLF administrations; **d** The progesterone levels of offspring from CTX-induced POF rats after rhLF administrations. Note: In each panel, labeling with the different letter in each column indicates significant differences between different groups (*P* < 0.05)

As shown in Fig. 9, the body weight and ovarian index of female offspring in all rhLF administration groups were significantly higher than the NC group (*P* < 0.05). Furthermore, the hormonal levels of estradiol and progesterone in all rhLF administration groups were significantly higher that the NC group (*P* < 0.05).

**Fig. 10:**
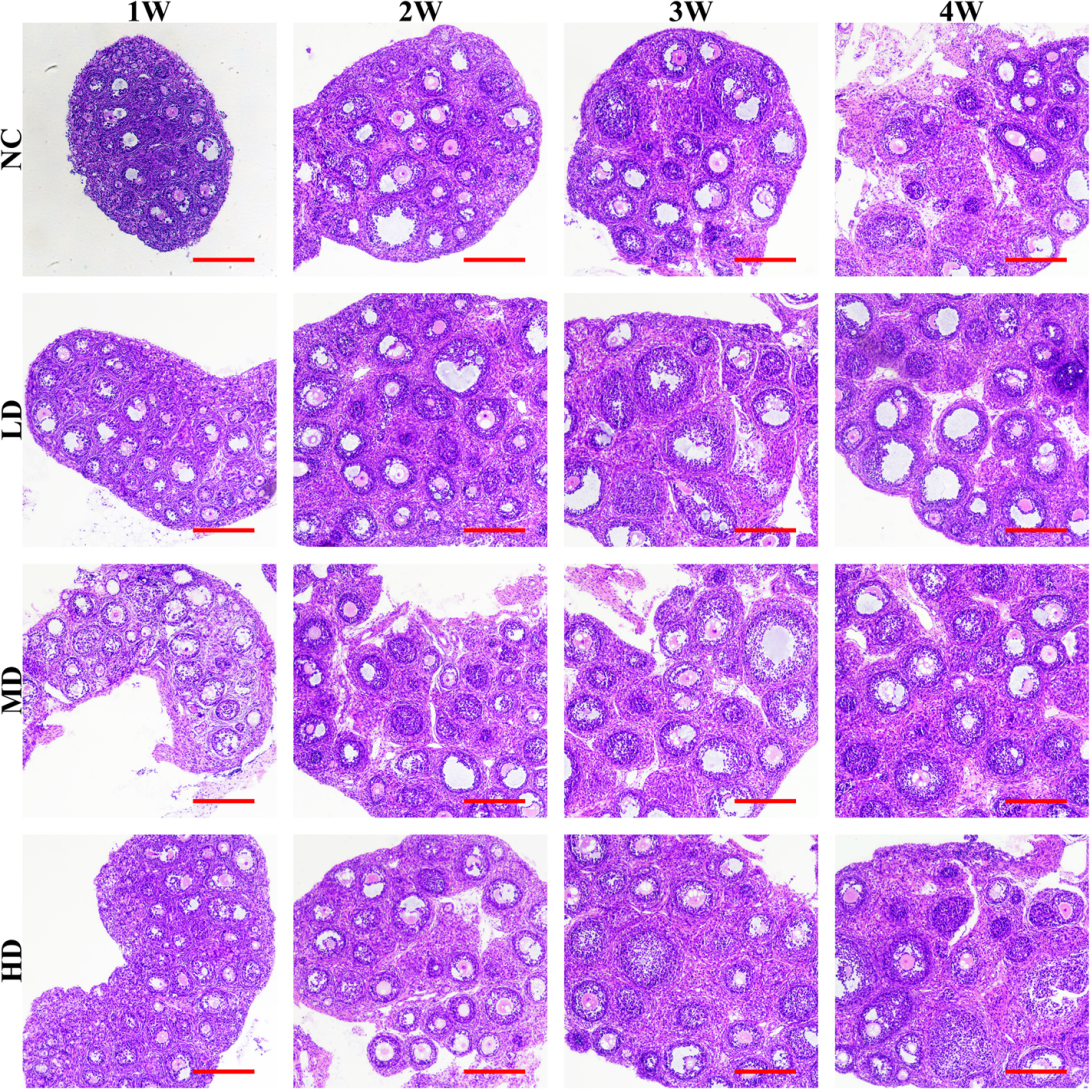
The representative HE staining results of offspring’s ovaries in different groups after rhLF administrations; Scale bar =200 μm

The HE staining results (Fig. 10) further confirmed that the oogenesis process in the offspring ovaries of all rhLF administration groups were up-regulated than that the NC group, however, the exact number of developing follicles and atretic follicles remains to be calculated.

## Discussion

Although the chemotherapy treatments effectively improved the prognosis of female cancer patients, the side effects and safety concerns of chemotherapy-induced POF including the risk of psychological distress, autoimmune disorders, infertility, ischemic cardiac diseases, sexual disorders, amenorrhea and osteoporosis still needs to be resolved [2, 79].

During the past decades, the clinical treatments for chemotherapy-induced POF patients have been developed rapidly with the treatments including hormone replacement therapy, stem cell therapy, ovary cryopreservation and donor-oocyte in vitro fertilization (IVF) technology already been applied for the POF-related symptoms [81], however, the clinical treatments of chemotherapy-induced POF patients remains unsatisfactory due to the complex pathogenesis of POF [11] or the increased risks for breast and endometrial cancers [33].

In our study, the effect and antioxidant related mechanisms of rhLF on the CTX-induced POF rats were analyzed to investigate the potential role of rhLF on the clinical treatment of cancer patients after CTX treatments.

Nowadays, many antioxidant supplements including vitamins, minerals, and polyphenols have been applied to reverse the oxidative stress-related damages and diseases caused by CTX treatments [18, 78, 84]. However, the safety of antioxidant active substances for the ovarian damages caused by oxidative stress needs further investigations.

In 2004, recombinant mouse LF receptor was found to be expressed in the various mouse tissues including brain, small intestinal epithelium, stomach, kidney and ovary [58]. In 2007, the existence of LF in murine follicular fluid and the correction between its concentration and embryonic development rates has been reported [75]. In 2008, the effect of bovine LF on the CTX-treated mouse ovaries was evaluated, and the results showed that the bovine LF administration rescued the CTX-induced ovarian damages with the gene expression level of *Adamts1* and *Sohlh1* and the protein expression of PCNA remarkably up-regulated [24]. In 2008, Stevenson et al reported that after oral supplements of bovine LF for 7 d, the total, helper and cytotoxic T-cell activation and hydrophilic antioxidant status of male volunteers were significantly promoted [44].

Furthermore, the antioxidant abilities of LF have been reported with the mitigation of liver damage, oxidative stress and hepatotoxicity caused by carbon tetrachloride treatments [17]. In addition, the hLF administration effectively suppressed the excessive iron accumulation caused by the 1-methyl-4-phenyl-1,2,3,6-tetrahydropyridine (MPTP) treatments and the up-regulation of divalent metal transporter (DMT1) and transferrin receptor (TFR), which subsequently improved the activity of antioxidant enzymes including SOD1 and GPX4 and decreased the ROS production levels [72].

In 2018, Ateya et al found that the LF administration significantly improved the levels of serum catalase, nitric oxide and GSH with a significantly down-regulated MDA level in a model of ossimi lambs, in addition, the immune-modulatory gene levels of *TNF-α*, *IL-1β*, *IL-6* and *IL-10* were significantly improved after LF administrations [15]. However, the antioxidant effect of rhLF against CTX-induced POF in rat ovaries and the potential target needs further investigations.

In our study, the antioxidant effects of rhLF against CTX-induced POF rats were confirmed with improved histological parameters, reduced lipid peroxidation process, increased antioxidant activities and decreased cellular apoptosis levels. And the in vitro assessment of rhLF against 4-HC-induced cytotoxicity further confirmed the main target of rhLF against CTX-induced POF damages was GCs. Furthermore, the fertility abilities of CTX-induced POF rats after rhLF administrations were resumed.

During the process of CTX treatments in vivo, the elevated oxidative stress in ovarian cells is significantly related with the apoptosis process of mice and rat GCs [46]. The histopathological staining results (including HE, Immunohistochemistry and Tunnel staining) in this study further indicated that the ovarian damages caused by CTX treatments were mainly located in rat GCs, instead of oocytes. Furthermore, the rhLF administration effectively reversed the apoptosis levels of rat GCs, which was confirmed by the observations that the gene expression levels of *Foxo3*, *Inhibin alpha* and *Fshr*, the hormonal levels of estradiol, progesterone and AMH (mainly secreted by GCs) after rhLF administration were significantly up-regulated.

During the oogenesis process in vitro, GCs maintains the ovarian local microenvironments for the development and maturation of oocytes via the secretion of mediators including follicle stimulating hormone (FSH), luteinizing hormone (LH), prostaglandins, chemokines and cytokines [12, 65]. The apoptosis of GCs has been treated as a leading contributor to POF in women [4, 30, 39]. During the entire process of oogenesis from embryonic stem cells (ESCs) and induced pluripotent stem cells (iPSCs), the GCs originated from the gonadal somatic cells play important roles on the growth and function of oocytes [22, 23], which further indicated the important roles of GCs during mammalian oogenesis.

The primary rat GCs was further applied to confirm the ameliorative effect of rhLF on POF damages. And the results showed that the rhLF treatments significantly protected the primary rat GCs from the 4-HC treatments via the inhibition of ROS production and the promotion of cellular proliferation, which was consistent with the in vivo results of rhLF administrations. In addition, the high expression levels of *Bax* after 4-HC treatments revealed the increased levels of cellular apoptosis, which was consistent with these previous studies about the exposure to chemotherapy drugs in vitro and high levels of GCs apoptosis [5, 14, 55, 82], and our results further confirmed the main target of rhLF treatments during POF was GCs. However, due to the complex in vivo microenvironments including oocytes and follicular cells (GCs, theca cells and stroma cells) in mammalian ovaries, the ameliorative target of rhLF on the POF damages needs more detailed in vitro investigations.

Furthermore, the estrous cycles and fertility abilities of POF rats after rhLF administrations were analyzed for the first time and the results showed that rhLF significantly reduced the abnormalities of estrous cycle caused by CTX treatments. In addition, the litter sizes of the CTX-induced POF rats after rhLF administrations were significantly up-regulated. Due to these altered maternal endocrine-metabolic environments and pregnancy-related complications of POF, the female fetus and long-term offspring health were adversely affected [13, 53], therefore, the effect of rhLF on the intergenerational transmission process of POF needs to be investigated to further confirm the safety of rhLF for clinical applications.

## Conclusion

This study provided direct evidences that the oral administrations of rhLF reduced the symptoms of CTX-induced POF by increasing the hormonal levels of estradiol, progesterone and AMH and decreasing the cellular apoptosis levels, while the ovarian antioxidant capacity and developing follicle numbers were significantly increased after rhLF administrations. In addition, our studies indicated that rhLF has a broad protective effect on rat ovarian functions with the potential target as GCs, which also indicates that rhLF could be applied as a promising supplement during the clinical treatment of POF.

## Methods

### Chemicals

Unless otherwise indicated, all chemicals, medium and supplements used in this study were purchased from Sigma Aldrich (Shanghai, China).

The freeze-dried powder of rhLF was gifted by Mr. Yunping Dai from the State Key Laboratory of Agricultural Biotechnology, China Agricultural University, Beijing, China.

The purity of rhLF was assessed using the reversed-phase high-performance liquid chromatography (RP-HPLC) by TSKgel protein C4–300 columns (TOSOH). For the preparation of rhLF solution, the powder of rhLF was dissolved with Dulbecco’s phosphate buffered saline (DPBS) solution and the concentration of rhLF applied in this study was optimized based on the human equivalent doses calculated by previous studies [25, 36].

### Animals and POF model induction

Eighty female Sprague Dawley (SD) rats (body weight as 200–220 g) were purchased from Beijing Vital River Laboratory Animal Technology Co., Ltd. (Beijing, China) and housed in the experimental animal room with controlled temperature (20–23 °C) and humidity (60 ± 5%) under standard 12-h light/dark cycles and free access to food and water.

After acclimatization for 1 week, the body weight and vaginal smear analysis of each rat were examined prior to drug administration to ensure the normal estrous cycle (defined as 4–6 d in length) and comparability of all experimental data among different administration groups [63].

For the induction of POF, all rats were intraperitoneally administered with 50 mg/kg CTX (C0768, Sigma Aldrich, Shanghai, China) on the first day and then with continuous 8 mg/kg CTX for 14 d according to the former studies [40, 76].

### rhLF administration

After CTX treatments, the rats were randomly assigned to four experimental groups (*n* = 20) as follows: the normal control group (NC group administrated with normal saline), low dose rhLF group (LD group, 150 mg/Kg), medium dose rhLF group (MD, 300 mg/Kg) and high dose rhLF group (HD, 450 mg/Kg), respectively, followed by the daily intragastric administration for 30 d.

### Assessment of estrous cycles

After the intragastric administration of rhLF, the estrous cycles of 10 rats from each group was checked by vaginal smear analyses [70] with the length of an estrous cycle calculated as the average number of days between two non-consecutive days [73].

### Hormonal assessment

To analysis the effect of rhLF administrations on the female reproductive functions of the CTX-induced POF rats, 10 rats at dioestrus from each group were randomly selected after estrous cycles assessment, and sacrificed by carbon dioxide with the body weight of each rat recorded and corresponding blood serum collected to analysis the hormonal levels of anti-Müllerian hormone (AMH), estradiol and progesterone with commercial ELISA kits according to the manufacturer’s instructions (E-EL-R3022 from Elabscience for AMH, PE223 from Beyotime for estradiol and PP773 from Beyotime for progesterone, Shanghai, China). To confirm the availability of experimental data, each sample was investigated in triplicate.

### Organ index analyses and HE staining

To analysis the effect of rhLF administrations on the ovarian development of the CTX-induced POF rats, after blood serum collection, the ovarian wet weights of each sacrificed rat were recorded with the ovarian index (ovarian weight/body weight× 100%) further analyzed, respectively.

After ovarian weight recorded, the ovaries (*n* = 5) of each sacrificed rat were individually kept in 4% paraformaldehyde solution (PFA, P1110, Solarbio, Beijing, China) for 24 h at room temperature for the following histological analyses.

After paraformaldehyde fixation, the ovaries were individually embedded in paraffin according to the department protocols, followed by 5 μm serial sections preparation and hematoxylin and eosin staining (HE) with commercial kit (G1120, Solarbio, Beijing, China) according to the manufacturer’s instructions [37].

### Histological analyses and follicle count

After HE staining, the number of follicles at different stages (primary, secondary, antral and atretic follicles) in every fifth section of each sacrificed rat was recorded with random start as detailed previously [47, 48, 83]. In addition, the numbers of corpus lutea in every fifth section of each sacrificed rat was counted.

To ensure the accuracy of follicle stages and numbers in different groups, the follicle count was conducted by a single well-trained ovarian histologist under a blinded fashion and two other members of the group periodically evaluated the random sections.

### Immunohistochemistry staining

To evaluate the effect of rhLF administrations on the proliferative abilities of follicles from the CTX-induced POF rats, Immunohistochemistry staining of Ki-67 protein was performed. Briefly, after dewaxing, gradient rehydration and antigen retrieval, the serial sections (*n* = 5) from each group were blocked with 5% bovine serum albumin (BSA, A8010, Solarbio, Beijing, China) in DPBS solution for 30 min at 37 °C. And the serial sections were incubated overnight at 4 °C with a rabbit anti-Ki67 antibody (with 1:300 diluted concentrations, 27,309–1-AP, Proteintech, Wuhan, China). After overnight incubation, the serial sections were rinsed with DPBS solution for three times (5 min for each time) and subsequently incubated with a goat anti-rabbit HRP secondary antibody (with 1:300 diluted concentrations, ZDR-5306, ZSGB-BIO, Beijing, China) at 25 °C for 2 h, respectively.

For the color reaction, the serial sections were incubated with fresh-prepared DAB substrate chromogen solution (DA1010, Solarbio, Beijing, China) at 37 °C for 3 min and re-stained with hematoxylin solution for 5 min. These stained sections were analyzed under a blinded fashion with a light microscope (CI-L, Nikon, Tokyo, Japan). Furthermore, the Ki-67 positive intensities in ovaries of different groups were analyzed by Image J based on the proportion of brown staining cells.

### Tunnel assays

To evaluate the effect of rhLF administrations on the disruption of DNA and cellular apoptosis levels in the follicles from the CTX-induced POF rats, Tunnel assays were conducted according to the manufacturer’s instructions. Briefly, after dewaxing and rehydration, 5 μm serial sections of each group were stained with the Tunnel apoptosis detection (FITC) kit (40306ES20, Yeasen, Shanghai, China). The morphometric analyses of Tunnel positive cells in the follicles of each group were performed and the apoptosis levels of each group were calculated with Image J based on the proportion of Tunnel positive cells (as apoptotic cells shown with green fluorescence).

### The assays of ovarian ROS, SOD, MDA and TAOC levels

To analysis the effect of rhLF administrations on the antioxidant abilities of the ovaries from CTX-induced POF rats, fresh ovaries (*n* = 5) from each group were collected for the assays of ovarian ROS, superoxide dismutase (SOD), malondialdehyde (MDA) and total antioxidative capacity (TAOC) levels. Briefly, after ovarian tissue homogenation, the levels of ROS, SOD, MDA and TAOC of different groups were detected by commercial assay kits (S0033S for ROS, S0109 for SOD, S0131 for MDA and S0121 for TAOC, Beyotime, Shanghai, China) according to the manufactures’ instructions.

### Ovarian PCR analyses

To analysis the effect of rhLF administrations on the ovarian gene expression levels of CTX-induced POF rats, total RNA of fresh ovaries (n = 5) from each group were extracted with Trizol solution (79,306, Gibco, Shanghai, China), respectively. The reversed synthesis of cDNA was carried out using a commercial Prime Script™ RT reagent kit (RR047A; Takara, Dalian, China), followed by Real-time PCR within a Thermo Scientific Pikoreal system by commercial kits (RR820A, Takara, Dalian, China). The qualities of PCR reactions were confirmed by the melting curves with all experiments performed in triplicate to confirm the data availability. The relative gene expression levels were calculated by the 2^-ΔΔCt^ method with the ubiquitously expressed *β-actin* gene used as internal controls [37].

The primers for reverse transcription PCR and Real-time PCR analyses were shown as follows.

- *Oct4*
  Forward primer-*CGAGGCCTTTCCCTCTGTTCCT*;
  Reverse primer-*TCTCTTTGTCTACCTCCCTTCCTTGC*;
- *Foxo3*
  Forward primer-*GGCAAAGCAGACCCTCAAACTGAC*;
  Reverse primer-*TGCCCACGATGGCAGGTCAC*;
- *Bmp15*
  Forward primer-*CCCTCCTTGCTGAAAACCCT*;
  Reverse primer-*TCAGCATGTACCTCAGGGGA*;
- *Gdf9*
  Forward primer-CAGGCTGGAGCCAGTGAAAA;
  Reverse primer-*TTAGGGGTCTCACTTCGCCT*;
- *Inhibin alpha*
  Forward primer-*ACAGGTGCCACCTGTGAGGA*;
  Reverse primer-*TGTCCCAAGGACACAGGCAC*;
- *Fshr*
  Forward primer-*TGCAAACTTGAAGCGGCAAATCTC*;
  Reverse primer-*CAAGACCCTGAGGATGTTGTACCC*;
- *Sod2*
  Forward primer-*CTGGCCAAGGGAGATGTTAC*;
  Reverse primer-*CAGCAACTCTCCTTTGGGT*;
- *Catalase*
  Forward primer-*GCGGATTCCTGAGAGAGTGG*;
  Reverse primer-*GAATCGGACGGCAATAGGAG*;
- *Gpx1*
  Forward primer-*CAGTTCGGACATCAGGAGAAT*;
  Reverse primer-*AGAGCGGGTGAGCCTTCT*;
- *Bax*
  Forward primer-*GAGGATGATTGCTGATGTGGATAC*;
  Reverse primer-*AGTTGAAGTTGCCGTCTGC*;
- *Bcl-2*
  Forward primer-*GACTGAGTACCTGAACCGGCATC*;
  Reverse primer-*CTGAGCAGCGTCTTCAGAGACA*;
- *β-actin*
  Forward primer-*GACTCATCGTACTCCTGCTTGCTG*;
  Reverse primer-*GGAGATTACTGCCCTGGCTCCTA*.

### Western blot

To analysis the effect of rhLF administrations on the ovarian protein expression levels of CTX-induced POF rats, the protein lysates of fresh ovaries (*n* = 5) from each group were extracted with a commercial protein extraction kit (DE101, TRANS, Beijing, China), respectively, followed by the protein content measurement by BCA kits (163–2086, BioRad, Beijing, China) according to manufacturer’s instructions.

The proteins lysates of each group were resolved on 10% SDS-PAGE gels (P1200, Solarbio, Beijing, China) and transferred to polyvinylidene fluoride (PVDF) membranes (IPVH00010, Millipore, Beijing, China), respectively. The PVDF membranes were blocked by 10% non-fat milk in Tris-HCl solution containing 0.1% Tween-20 (TBST) and separately incubated with the following primary antibodies at 4 °C overnight: a rabbit anti-Bax antibody (with 1:1000 diluted concentration, 50,599–2-lg, Proteintech, Wuhan, China), a rabbit anti-Bcl-2 antibody (with 1:1000 diluted concentration, 12,789–1-AP, Proteintech, Wuhan, China), a rabbit anti-Caspase 3 antibody (with 1:1000 diluted concentration 19,677–1-AP, Proteintech, Wuhan, China) and a rabbit anti-GAPDH antibody (with 1:1000 diluted concentration, ab8245, Abcam, Shanghai, China) as loading controls, respectively.

The membranes were washed three times with TBST solution and incubated with goat anti-rabbit HRP secondary antibodies (with 1:1000 diluted concentration, ZDR-5306, ZSGB-BIO, Beijing, China) at room temperature for 1 h, respectively. The blots were visualized with enhanced chemiluminescence solutions (ECL, W1001, Promega, Beijing, China) and recorded within a ChampChemi 610 Plus system. Furthermore, the protein expression levels normalized to GAPDH were analyzed by Image J.

### Isolation and culture of primary rat granulosa cells

The isolation and culture of primary rat granulosa cells (GCs) were conducted as previously reported [6, 61]. Briefly, the immature female rats (21 d old) were injected intraperitoneally with 10 IU/mL pregnant mare serum gonadotropin (PMSG, Sansheng, Ningbo, China) and sacrificed by cervical dislocation after 48 h. The bilateral ovaries were immediately collected and washed in DPBS solution supplemented with 100 U/mL penicillin/streptomycin antibiotics (P/S, 15070063, Invitrogen, China). After the removal of surrounding fat tissues, the ovaries were placed in the ice-cold DPBS solution and punctured with 27-gauge needles. The ovaries were then digested with hyaluronidase solution (H3605, Sigma Aldrich, Shanghai, China), followed by the collection of primary rat GCs. after cell counting, the primary rat GCs were cultured in 24 well plates (Corning, Beijing, China) at a density of 5 × 10^4^ cells/ well in a humidified atmosphere of 5% CO_2_ and 95% air at 37 °C. For the following studies, the 24 well plates were pre-plated with sterile cover-slips. The culture medium for primary rat GCs was the DMEM/F12 medium (11,320,033, Gibco, Shanghai, China) supplemented with 15% FBS (10,091,148, Gibco, Shanghai, China) and 100 U/mL P/S.

### Rat GCs treatments and experimental group settings

The activity of CTX requires a 4-hydroxylation reaction in the liver tissues, leading to the incapable applications of CTX in vitro. 4-hydroperoxy cyclophosphamide (4-HC), as the active in vitro metabolite of CTX, was applied for the following treatments of rat GCs [59, 80].

During the primary rat GCs culture in vitro, 100 μg/mL 4-HC solution (gifted by Mrs. Xiulan Su from the Key Laboratory of Medical Cell Biology, Clinical Medicine Research Center, the Affiliated Hospital of Inner Mongolia Medical University) was supplemented to the culture medium of primary rat GCs based on the former studies about the rat GCs toxicity of 4-HC [80]. During the 4-HC treatments for 24 h, the culture medium of primary rat GCs was also supplemented with DPBS (the negative control group, rhLF 0 group), 100 μg/mL rhLF (rhLF 100 group), 200 μg/mL rhLF (rhLF 200 group) and 300 μg/mL rhLF (rhLF 300 group), respectively, followed by the cell culture in a CO_2_ incubator (37.0 °C, 5% CO_2_).

### ROS assay of rat GCs

To analysis the ameliorative effect of rhLF treatments on the antioxidant abnormalities of rat GCs caused by 4-HC in vitro, the ROS production levels of primary rat GCs after drug treatments were detected with ROS assay kits (S0033, Beyotime, Shanghai, China) according to the manufacturer’s instructions.

Briefly, the primary rat GCs pre-plated on sterile cover-slips were washed three times with DPBS solution and incubated with 10 μM dichlorofluorescein diacetate (DCFH-DA, S0033, Beyotime, Shanghai, China) for 30 min at 37 °C, respectively. After DCFH-DA incubation, the cells were individually re-stained with 10 μg/mL Hoechst 33432 solutions (C0030, Solarbio, Beijing, China) for 5 min at 37 °C. The cellular fluorescence staining densities in different groups were analyzed by an inverted fluorescence microscopy (Ti, Nikon, Tokyo, Japan).

### EdU staining of rat GCs

To analysis the ameliorative effect of rhLF treatments on the abnormal proliferation of rat GCs caused by 4-HC in vitro, the proliferation of rat GCs after drug treatments were detected with EDU (5-ethynyl-2′-deoxyuridine) assay kits (C0071S, Beyotime, Shanghai, China) according to the manufacturer’s instructions.

Briefly, the primary rat GCs pre-plated on sterile cover-slips were washed three times with DPBS solution and incubated with EdU solution for 2 h, followed by the re-staining with 10 μg/mL Hoechst 33432 solutions for 5 min at 37 °C. After cover-slip sealing, the cellular fluorescence staining densities in different groups were analyzed by an inverted fluorescence microscopy.

### PCR analyses of rat GCs

To analysis the ameliorative effect of rhLF treatments on the abnormal gene expression levels of rat GCs caused by 4-HC in vitro, the expression levels of *Inhibin alpha*, *Fshr*, *Sod2*, *Catalase*, *Gpx1, Bax* and *Bcl-2* in rat GCs after drug treatments were detected with PCR analyses according to the methodologies in the section of ovarian PCR analyses.

### Fertility assessment after rhLF administrations

The fertility assessment was performed to assess the potential effects of rhLF on the fertilities of CTX-induced POF rats. In order to avoid the risk of CTX-related fetal malformations [43], 4 weeks after rhLF administrations, 10 rat of each group were individually paired with normal male rats (12 weeks old) of proven fertility at a 1:1 ratio, followed by the mating examination (appearance of the vaginal plug). On the day when copulation was confirmed, the successful mating days were recorded with the female rat of each group housed individually. On postnatal day (PND) 0, the gestation length of each group and the offspring data (number of pups per litter and pup weight) were recorded.

Every other week, three female offspring of each group were humanely sacrificed by carbon dioxide with the body weight recorded and hormonal levels of estradiol and progesterone analyzed. Furthermore, the ovaries of each sacrificed offspring were collected, followed by the analyses of ovarian index, preparation of paraffin sections and HE staining.

### Statistical analyses

In this study, the results were expressed as means±SEM. One-way analyses of variance (ANOVA) with IBM SPSS statistic’s software 19.0 were applied to analysis the experimental data among different groups, followed by the LSD comparison post-test. *P* < 0.05 was considered as significant.

## Data Availability

We declared that materials described in the manuscript, including all relevant raw data, will be freely available to any scientist wishing to use them for non-commercial purposes, without breaching participant confidentiality.

## Abbreviations

POF: Premature ovarian failure
AMH: Anti-Müllerian hormone
MDR: Multidrug resistance
ROS: Reactive oxygen species
CTX: Cyclophosphamide
RP-HPLC: Reversed-phase high-performance liquid chromatography
DPBS: Dulbecco’s phosphate buffered saline
HE: Hematoxylin and eosin staining
SOD: Superoxide dismutase
MDA: Malondialdehyde
TAOC: Total antioxidative capacity
PVDF: Polyvinylidene fluoride
TBST: Tris-HCl solution containing 0.1% Tween-20
PND: Postnatal day
ANOVA: One-way analyses of variance
IVF: In vitro fertilization
MPTP: 1-methyl-4-phenyl-1,2,3,6-tetrahydropyridine
DMT1: Divalent metal transporter
TFR: Transferrin receptor
FSH: Follicle stimulating hormone
LH: Luteinizing hormone
ESCs: Embryonic stem cells
iPSCs: Induced pluripotent stem cells
GCs: Granulosa cells
PMSG: Pregnant mare serum gonadotropin
P/S: Penicillin/streptomycin antibiotics
4-HC: 4-hydroperoxy cyclophosphamide
DCFH-DA: Dichlorofluorescein diacetate
EDU: 5-ethynyl-2′-deoxyuridine
